# Will Quantum Topology Redesign Semiconductor Technology?

**DOI:** 10.3390/nano15090671

**Published:** 2025-04-28

**Authors:** Giuseppina Simone

**Affiliations:** 1 Dipartimento di Ingegneria Chimica, University of Napoli Federico II, Piazzale Tecchio 80., 80125 Napoli, Italy; giuseppina.simone@unina.it; 2 Department of Physics, New Uzbekistan University, Tashkent 100007, Uzbekistan

**Keywords:** semiconductor, topology, non-hermitian semiconductor

## Abstract

Semiconductors underpin modern technology, enabling applications from power electronics and photovoltaics to communications and medical diagnostics. However, the industry faces pressing challenges, including shortages of critical raw materials and the unsustainable nature of conventional fabrication processes. Recent developments in quantum computing and topological quantum materials offer a transformative path forward. In particular, materials exhibiting non-Hermitian physics and topological protection, such as topological insulators and superconductors, enable robust, energy-efficient electronic states. These states are resilient to disorder and local perturbations, positioning them as ideal candidates for next-generation quantum devices. Non-Hermitian systems, which break traditional Hermitian constraints, have revealed phenomena like the skin effect, wherein eigenstates accumulate at boundaries, violating bulk-boundary correspondence. This effect has recently been observed in semiconductor-based quantum Hall devices, marking a significant milestone in condensed matter physics. By integrating these non-Hermitian topological principles into semiconductor technology, researchers can unlock new functionalities for fault-tolerant quantum computing, low-power electronics, and ultra-sensitive sensing platforms. This convergence of topology, quantum physics, and semiconductor engineering may redefine the future of electronic and photonic devices.

## 1. An Overview of the Topological Quantum Materials and Trend

**Figure 1:**
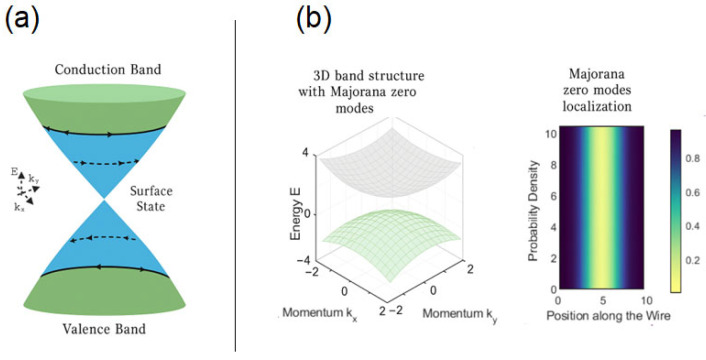
Schematic of some quantum optical materials. (**a**) Quantum insulator. Valence and conduction bands characterize the energy modes together with the topological surface states. (**b**) Topological Superconductor with Majorana Zero Modes: 3D Band Structure Visualization (**left**) and Real-Space Probability Density given by a heatmap to show how Majorana zero modes localized at the ends of geometry (e.g., a wire).

The semiconductor industry is currently facing several pressing challenges that threaten to hinder technological progress. One of the greatest issues is the global shortage of critical materials, such as gallium, indium, and high-purity silicon, which are essential for semiconductor fabrication. The increasing demand for these materials, coupled with geopolitical disruptions of the supply chain, has led to significant volatility of prices and manufacturing delays. Furthermore, sustainability concerns are becoming more pronounced as traditional semiconductor production processes consume vast amounts of energy, utilize toxic chemicals, such as perfluorinated compounds, and generate hazardous electronic waste. The environmental footprint of semiconductor fabrication is a growing issue that requires new materials and methodologies to improve energy efficiency and reduce resource dependence. Addressing these challenges requires innovative solutions, and quantum topological materials have emerged as a promising alternative. Their unique electronic properties, including *sans*-dissipation edge states and intrinsic fault tolerance, could potentially revolutionize semiconductor technology, leading to more energy-efficient and sustainable computational paradigms. Topological quantum materials are structures renowned for their symmetrically protected and remarkably mobile electronic states [1,2]. Among these, topological insulators are an extraordinary category that defies simple classification as either insulators, semiconductors, or metals. These materials possess a band gap that separates the valence and conduction bands on a subatomic scale, while at their surface dynamic metallic states, known as topological surface states, these bands are bridged, creating a striking interplay of electronic phenomena [3]. Topological superconductors, another class of topological quantum materials, possess the ability to host Majorana-bound states, e.g., Majorana zero modes [4] (Figure 1).

The quasiparticle excitations, which emerge at the boundaries of both one-dimensional and two-dimensional systems, embody the intriguing properties of Majorana fermions [5]. Their creation and annihilation operators are not functional; they are strikingly identical and self-adjoint, giving rise to a fascinating phenomenon wherein these entities are their antiparticles, blurring the lines between matter and antimatter, and holding a memory of the relative positions over time. Therefore, Majorana fermions, while being extraordinary particles that defy convention by being their antiparticles, emerge within the unique realm of topological superconductors, distinguishing them from ordinary metals.

In the everyday world of typical metals and insulators, quasiparticles, such as electrons and holes, carry a specific electrical charge, while their antiparticles bear the opposite charge. This fundamental property makes the formation of Majorana fermions exceptionally unlikely in these materials, underscoring the rare and fascinating nature of topological superconductors. These materials hold significant promises for transforming quantum computation by acting as novel qubits. To better understand the significance of Majorana fermions in quantum computing, it is useful to consider an analogy rooted in particle physics and quantum mechanics. In classical fermionic systems, every particle has a distinct antiparticle with an opposite charge, much like an electron and a positron. However, in the case of Majorana fermions, a single quasiparticle simultaneously behaves as its antiparticle, meaning that it can encode quantum information in a fundamentally different way. This unique property is analogous to a topological knot: just as a knotted loop preserves its topology unless it is cut, Majorana fermions store quantum information in a non-local manner, making them highly resistant to decoherence and local disturbances. This robustness is what renders them ideal candidates for topologically protected quantum computation. Topological superconductors provide a pathway to inherently fault-tolerant quantum technologies, incorporating error correction directly into hardware [6]. The non-local characteristics of Majorana-bound states enhance their resilience against local noise, a primary source of errors in existing quantum processors. This resistance to local noise has the potential to streamline the design of scalable quantum systems. In addition to enabling fault-tolerant quantum memories through their non-locality [7], the non-Abelian statistics of Majorana-bound states could also facilitate the implementation of fault-tolerant quantum logic gates.

Since the conceptual foundations of quantum computing and Majorana-bound states were established in the first decade of this millennium, there has been a growing focus on their experimental exploration. To investigate and harness the unique properties of Majorana-bound states, hybrid superconductor–semiconductor structures have been developed [8], with initial signatures of their behavior demonstrated as early as [9]. At UC Santa Barbara, researchers successfully realized Majorana zero modes by precisely positioning an indium arsenide (InAs) semiconductor nanowire adjacent to an aluminum (Al) superconductor. This hybrid structure enabled superconductivity to be induced in the nanowire via the proximity effect. Crucially, the experimental setup required ultra-low temperatures (below 50 mK) and a magnetic field above a critical threshold (typically >0.5 T) to open a Zeeman gap. Spin–orbit coupling within the InAs nanowire further enabled the formation of a topological superconducting phase and Majorana zero modes emerged at the ends of the nanowire, localized and protected by the topological energy gap. In this case, key technical challenges included achieving epitaxial growth of Al on InAs to ensure a clean interface, minimizing disorder along the nanowire, and tuning gate voltages to control the chemical potential. Achieving strong proximity coupling in nanowire–superconductor devices demands meticulous control over fabrication parameters, with performance highly sensitive to deviations. The quality of the interface is paramount: epitaxial alignment between the semiconductor nanowire (e.g., InAs) and superconductor (e.g., Al) must achieve atomic-scale precision to minimize disorder, as even minor misalignment (>1–2 nm) can disrupt induced superconductivity. Material purity further dictates coupling strength, necessitating ultra-high vacuum deposition and in-situ cleaning to eliminate interfacial oxides or defects. The proximity effect itself hinges on optimizing the superconductor thickness (typically 5–15 nm) to balance Cooper pair penetration with bulk superconducting properties, while low-resistance ohmic contacts (<1 kΩ) preserve topological gap integrity. Device performance degrades sharply with interfacial disorder or strain, which can suppress the topological gap or obscure Majorana zero modes. Thermal stability during fabrication is equally critical, as temperatures exceeding 150 °C may cause material interdiffusion. Experimental studies demonstrate that suboptimal interfaces can preclude Majorana signatures altogether, underscoring the need for nanoscale fabrication control [8]. Recent advances in cryogenic lithography and selective-area growth aim to address these challenges, though reproducibility remains a hurdle for scalable deployment. The stability and detection of Majorana zero modes in hybrid superconductor–semiconductor nanowire systems are significantly hampered by environmental decoherence. The parity conservation necessary for Majorana-based topological qubits can be compromised by thermal fluctuations that activate quasiparticles across the superconducting gap. The functioning of the device is restricted to dilute refrigerator temperatures, usually below 50 mK, due to these conditions. The induced topological phase might become unstable due to voltage variations on the gates or in the superconductor caused by electromagnetic interference from high-frequency noise. Experimental techniques use low-pass filtering at various temperature stages in the dilution refrigerator and significant electromagnetic shielding of the measuring apparatus to lessen these effects. Devices are also designed with superconducting leads and capacitive shunting to minimize coupling to external noise sources. Charge noise mitigation is achieved through material purification and careful dielectric engineering to reduce trap densities near the active device region. Dynamical decoupling and parity readout schemes using quantum dots or transmon qubits have also been proposed to enhance robustness. Despite these efforts, maintaining coherent parity lifetimes beyond a few hundred microseconds remains an active area of research, underscoring the need for improved materials and noise mitigation techniques to enable fault-tolerant Majorana-based quantum computing.

Despite these problems, topological quantum materials exhibit unique properties that distinguish them from conventional materials. Unlike traditional conductors or semiconductors, topological insulators support edge states that are robust against scattering by disorder or defects, due to topological protection. Moreover, in topological superconductors, quasiparticles known as Majorana-bound states obey non-Abelian statistics, making them promising candidates for fault-tolerant quantum computation. These states are immune to local perturbations and enable quantum information to be stored in a nonlocal fashion, significantly reducing the need for complex error correction. Additionally, the dissipation*less* nature of edge or surface states offers energy efficiency and operational stability for low-power devices and robust sensing applications.

## 2. The Topological Aspect of Modern Quantum Computing

### 2.1. Non-Hermitian Physics and Skin Effects

Hermiticity is a fundamental property in quantum mechanics, ensuring energy conservation and governing the evolution of closed systems. However, in open systems where interactions with external environments are significant, Hermiticity is often violated, giving rise to non-Hermitian dynamics. These dynamics are characterized by complex energy spectra and non-unitary evolution, which can lead to novel physical phenomena not observed in traditional Hermitian systems. In recent years, the study of non-Hermitian physics has surged, driven by its potential to revolutionize our understanding of quantum systems and its applicability across diverse fields [10,11,12,13].

This includes open quantum systems, where decoherence and dissipation play critical roles, semiconductor systems with non-Hermitian interactions, and classical wave systems exhibiting gain or loss mechanisms. One of the most intriguing developments in this domain is the discovery of the non-Hermitian skin effect, a phenomenon that challenges conventional wisdom in condensed matter physics and photonics. The non-Hermitian skin effect arises in non-Hermitian systems with open boundary conditions, where an extensive number of eigenstates become localized at the boundaries, in stark contrast to the extended Bloch modes typically observed under periodic boundary conditions [14]. This localization is a direct consequence of the non-Hermitian nature of the system, which breaks the conventional bulk-boundary correspondence, a cornerstone of Hermitian topological physics. In one-dimensional lattices, the non-Hermitian skin effect is closely tied to a topological invariant known as the point-gap winding number, which quantifies the winding of the complex energy spectrum around a reference point in the complex plane. The invariant provides a robust framework for understanding the topological properties of non-Hermitian systems.

The implications of the non-Hermitian skin effect and the non-Hermitian topology are profound [15]. The systems with a non-Hermitian nature exhibit exceptional sensitivity to boundary conditions and perturbations, making them ideal candidates for high-precision sensors and amplifiers. In addition, the ability to control and manipulate skin modes opens new avenues for designing advanced photonic devices, such as light funnels and waveguides, with unprecedented efficiency and functionality. The interplay between non-Hermitian dynamics and topological phenomena also offers a rich platform for exploring novel quantum states and phase transitions, potentially paving the way for breakthroughs in quantum computing and information processing. Beyond photonics, non-Hermitian physics holds immense promise in semiconductor technology. In semiconductor devices, dissipation and non-reciprocal transport properties can be harnessed to engineer novel functionalities, such as ultra-sensitive detectors and robust electronic states that are resilient to disorder. By leveraging non-Hermitian topological effects, researchers can design semiconductor structures with asymmetric charge transport, which could enhance the performance of transistors, diodes, and other critical components. Furthermore, the incorporation of non-Hermitian principles into semiconductor platforms can lead to new paradigms in optoelectronic devices. For example, the control of gain and loss in semiconductor lasers can improve their coherence and efficiency, while non-Hermitian engineered materials may enable directional energy transfer with minimal dissipation. These advancements have the potential to redefine the architecture of future semiconductor circuits, providing new opportunities for energy-efficient electronics and next-generation communication technologies. By integrating non-Hermitian physics with semiconductor engineering, researchers can unlock unprecedented functionalities, driving innovation in both classical and quantum electronic systems. In summary, non-Hermitian physics, particularly the non-Hermitian skin effect, represents a paradigm shift in our understanding of quantum and classical systems. By leveraging the unique properties of non-Hermitian systems, researchers are unlocking new possibilities for technological innovation and deepening our comprehension of the fundamental principles governing physical reality.

### 2.2. Non-Hermitian Quantum Semiconductor

Non-Hermitian topology has been experimentally demonstrated in quantum condensed-matter devices. Observations of non-Hermitian phenomena have largely been limited to systems, such as ultracold atoms, optical platforms, and classical metamaterials. However, a groundbreaking study has achieved the first direct observation of non-Hermitian topology in a quantum condensed-matter system. This was accomplished using a multiterminal quantum Hall device, where the non-reciprocity of quantum Hall edge states, rather than traditional gain and loss mechanisms, was harnessed to reveal non-Hermitian effects. The team designed an innovative quantum Hall device based on a two-dimensional electron gas ring fabricated from an aluminum gallium arsenide/gallium arsenide semiconducting heterostructure. Interestingly, the device features a ring-shaped geometry with multiple arms distributed along its external perimeter, each connected to the inner ring via ohmic contacts. This design enabled one of the first explorations of non-Hermitian topological effects in a semiconductor platform, bridging the gap between quantum mechanics and condensed-matter physics [16]. The choice of aluminum gallium arsenide/gallium arsenide was motivated by its superior electronic properties; alternative semiconductor platforms have been explored for realizing non-Hermitian topological effects. However, there is growing interest in alternative semiconductor platforms that could support similar phenomena, particularly in contexts where integration, scalability, or photonic implementations are desired. Silicon photonics has emerged as a compelling platform for non-Hermitian physics, particularly in systems utilizing coupled waveguides with engineered gain and loss. While silicon itself is an indirect bandgap material and not ideal for electronic topological transport, its mature fabrication ecosystem and low optical losses in the near-infrared regime make it well-suited for simulating non-Hermitian Hamiltonians in a photonic context. Recent work has demonstrated parity-time symmetric systems and topological edge modes in silicon-based lattices, albeit with limited dynamic tunability compared to GaAs-based systems. III-nitride semiconductors, such as GaN/AlN, provide an alternative with wide bandgaps and strong built-in polarization fields. These materials offer high breakdown voltages and thermal stability, which are advantageous for high-power applications, though their relatively lower electron mobilities (1000 cm^2^/V s) and more complex growth dynamics limit their applicability to precision topological transport. Compared to aluminum gallium arsenide/gallium arsenide, these alternative systems trade off performance metrics, such as mobility and interface quality, for benefits in integration and versatility. While they may not yet match the performance of GaAs-based devices in electronic non-Hermitian regimes, they open valuable avenues for realizing topological effects in scalable and application-relevant architectures.

**Figure 2:**
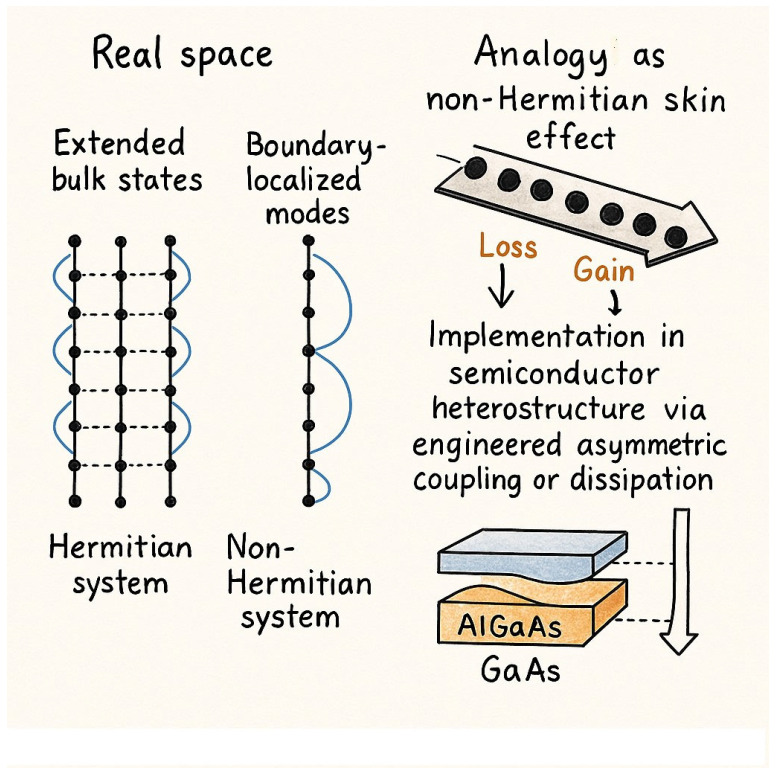
Illustration of the non-ergodic skin effect in a topological non-Hermitian system. Left: In an ergodic (Hermitian) system, wave functions are uniformly distributed across the entire sample. Right: In a non-Hermitian system exhibiting the non-ergodic skin effect, eigenstates localize at the boundaries, violating conventional bulk-boundary correspondence. This boundary accumulation of modes enables robust edge-state control in semiconductor-based devices under engineered non-Hermitian conditions.

The semiconductor-based quantum Hall ring allows for the experimental realization of two distinct configurations of the Hatano–Nelson model, one of the simplest and most fundamental examples of non-Hermitian topology [17]. Besides, the device permits continuous tuning between open boundary conditions and periodic boundary conditions, offering a versatile platform to study the transition between localized and extended states. This tunability is critical for probing the unique properties of non-Hermitian systems, particularly the non-Hermitian skin effect, which manifests as the exponential localization of eigenstates at the system’s boundary under open boundary conditions. A key insight is that the non-Hermitian skin effect, akin to the quantized Hall conductance in the quantum Hall effect, serves as a directly observable transport property of the semiconductor device. Unlike conventional approaches that require the determination of the conductance matrix or its numerical diagonalization, the skin effect can be inferred from the exponential localization of all eigenvectors of the conductance matrix at one boundary. This localization gives rise to robust and distinctive transport signatures, characterized by exponential profiles in current and voltage distributions. The transport signatures, on the other hand, remain consistent across a wide range of magnetic fields, are independent of the initial current configurations, and are exclusively observed under open boundary conditions. Moreover, the robustness of these signatures, rooted in the non-Hermitian topology of the system, underscores the device’s stability and reliability. This discovery holds significant promise for the advancement of semiconductor technology, as it introduces novel ways to manipulate electron transport properties for next-generation electronic and quantum devices. The ability to engineer and control non-Hermitian topological effects in semiconductor-based systems could lead to new methods for enhancing device performance, particularly in areas, such as ultra-low-power electronics, high-sensitivity sensors, and topologically protected signal processing. Unlike conventional semiconductor technologies, which rely on Hermitian physics, leveraging non-Hermitian principles allows for the design of devices that can exploit robust transport properties, even in the presence of disorder and imperfections. The integration of non-Hermitian physics into semiconductor platforms paves the way for breakthroughs in quantum information processing. The exponential localization of eigenstates observed in non-Hermitian systems may be harnessed for the development of fault-tolerant quantum circuits, where signal integrity is preserved against environmental fluctuations. Semiconductor devices incorporating non-Hermitian topology could serve as key components in optical and electronic signal amplification, offering higher efficiency and stability in comparison to traditional amplifiers. It is interesting that by merging non-Hermitian physics with established semiconductor fabrication techniques, researchers can develop a new class of devices that capitalize on unique quantum transport properties, ultimately transforming the landscape of quantum and classical semiconductor technologies. This discovery not only advances our understanding of non-Hermitian physics in quantum condensed-matter systems but also opens new avenues for designing robust quantum devices with applications in precision sensing, signal amplification, and quantum information processing. The work represents a significant milestone in the intersection of non-Hermitian physics and semiconductor technology. By demonstrating non-Hermitian topology in a quantum Hall device, the researchers have established a new paradigm for exploring and harnessing non-Hermitian phenomena in solid-state systems. Figure 2 summarizes some important differences between the classical Hermitian and non-Hermitian systems.

### 2.3. Topological Quantum Technology Impact on Semiconductors

The topological skin effect ensures that currents flowing between different contacts on a quantum semiconductor remain robust against impurities or external perturbations. This remarkable property significantly enhances the appeal of topological devices in the semiconductor industry, as it eliminates the need for ultra-high material purity, a requirement that traditionally drives up manufacturing costs.

Several studies have quantitatively benchmarked the robustness of topological materials against disorder. For instance, topological insulators and superconductors exhibit quantized conductance (e^2^/h) even in the presence of disorder strengths up to 10–20% of the bandwidth [18]. In contrast, similar levels of disorder in conventional semiconductor systems lead to Anderson localization and a drastic reduction in carrier mobility. Moreover, simulations on quantum spin Hall systems show that topological edge states can tolerate up to 5–10% vacancy defects without closing the bulk gap or disrupting edge transport [19]. These results provide quantitative support for the assertion that topological devices possess enhanced tolerance to impurity scattering relative to conventional semiconductors.

By mitigating the sensitivity to material defects, topological devices offer a cost-effective pathway for producing high-performance electronics. A novel topological quantum device, with a diameter of approximately 0.1 mm, has been developed, showcasing substantial scalability potential. This device leverages a carefully designed arrangement of materials and contacts on an aluminum gallium arsenide semiconductor substrate. When subjected to ultra-cold temperatures and a strong magnetic field, the system exhibits the topological skin effect. This phenomenon arises from the interplay between non-Hermitian physics and the quantum Hall effect, creating a unique regime where boundary-localized states dominate the system’s behavior. The induced topological phase is characterized by currents that are inherently protected from scattering, ensuring robust and efficient charge transport. Further investigation into this effect is essential to fully unlock its potential for technological advancements. However, while the reduction in material purity requirements contributes to cost-effectiveness at the material level, several key challenges remain for scaling up topological quantum devices to industrial levels. Firstly, the operation of current prototypes still relies on cryogenic temperatures (typically below 1 Kelvin) and high magnetic fields, which necessitate expensive cooling systems and complex infrastructure not yet suitable for widespread deployment. Secondly, the fabrication of these devices involves nanoscale precision in the alignment and patterning of heterostructures, requiring advanced lithography techniques that may not yet be optimized for high-throughput production. Finally, maintaining the coherence and stability of topological states over large arrays or integrated circuits remains an open research problem, as small perturbations or thermal fluctuations at scale could degrade device performance. Moreover, integrating these quantum systems with existing CMOS-based technologies presents both physical and architectural challenges that need to be overcome before commercial viability is achieved. By deepening our understanding of the underlying mechanisms, researchers can optimize the design and functionality of topological devices, boosting the integration into next-generation semiconductor technologies. These devices hold promise for applications that require high efficiency, reliability, and scalability, such as quantum computing, low-power electronics, and advanced sensing systems.

As mentioned above, the stability of topological skin effect phases presents a complex interplay between intrinsic topological protection and extrinsic operational factors. While *sans*-dissipation edge states demonstrate inherent robustness against static disorder, extended operation reveals notable challenges, including hysteresis effects and material aging. Experimental studies of Aluminum gallium arsenide/Aluminium gallium arsenide quantum Hall rings have documented hysteresis in boundary-localized states during cyclic magnetic field variations, attributed to delayed edge current relaxation during phase transitions. Long-term stability is further compromised by material degradation mechanisms, where cryogenic cycling and sustained magnetic fields induce interfacial stress in heterostructures, like Indium Arsenide /Aluminium nanowires, accelerating defect migration. Concurrently, progressive charge trap accumulation in dielectric layers degrades topological gap coherence, with parity lifetimes decaying to approximately 100 µs over weeks of continuous operation. Mitigation approaches currently under investigation include encapsulation with hexagonal boron nitride to inhibit interfacial oxidation and dynamic gate voltage tuning to compensate for charge drift. These findings highlight critical reliability considerations that must be addressed before topological devices can achieve industrial viability, particularly for applications requiring sustained operation under variable environmental conditions. The development of accelerated aging protocols and fault-tolerant architectures remains an active area of research focus in the field.

While the current experiments require cryogenic temperatures and strong magnetic fields to achieve the quantum Hall regime, several engineering approaches have been proposed to enable room-temperature operation. One key approach involves leveraging two-dimensional materials, such as graphene, which exhibits exceptionally high carrier mobilities and a linear dispersion relation near the Dirac point. Graphene has demonstrated quantum Hall plateaux at temperatures approaching 300 K when subjected to magnetic fields exceeding 30 T. However, the required magnetic field strength remains impractically high for most device applications. Alternative strategies include the use of magnetic topological insulators exhibiting the quantum anomalous Hall effect, wherein intrinsic magnetization can replace the need for external fields. In Cr- or V-doped (Bi,Sb)_2_Te_3_ thin films, quantized Hall conductance has been observed without an external magnetic field, although still at milli-Kelvin temperatures. The primary limitation lies in the small exchange-induced gap (tens of µeV), which is easily overcome by thermal fluctuations at higher temperatures. Furthermore, recent developments in Moiré superlattices and strain engineering have also shown potential for enhancing bandgap scales and modifying topological characteristics to stabilize topological phases under thermal perturbations. However, a fundamental barrier remains: the energy separation between Landau levels or topological gaps must exceed *k_B_T* at room temperature (25 meV), which is currently only achievable under extreme magnetic or material conditions. While these approaches collectively point toward possible engineering pathways to higher temperature operation, overcoming the thermal and field-scale constraints represents a fundamental and ongoing challenge in realizing practical, room temperature quantum Hall devices.

## 3. Conclusions

In summary, the actual state-of-the-art marks a groundbreaking achievement at the intersection of non-Hermitian physics and semiconductor technology. In fact, by demonstrating non-Hermitian topology in a quantum Hall device, the researchers have established a new framework for exploring and exploiting non-Hermitian phenomena in solid-state systems. This breakthrough not only advances fundamental scientific understanding but also opens up exciting possibilities for practical applications. The integration of topological quantum effects into semiconductor devices represents a transformative step forward, offering the potential to revolutionize the efficiency, robustness, and scalability of future electronic and quantum technologies.

To fully unlock this potential, several concrete research and engineering challenges must be addressed. Current demonstrations rely on extreme operational conditions, such as cryogenic temperatures (below 1 K) and strong magnetic fields (>5 T)—which are not practical for commercial systems. Future work must focus on realizing topological phases under ambient or near-ambient conditions, possibly through intrinsic magnetic materials, engineered spin-orbit coupling, or electric-field-induced band structure tuning. Scalability is another major challenge. Existing fabrication methods often depend on high-precision, low-throughput techniques, like electron-beam lithography and molecular beam epitaxy. To move toward industrial applications, scalable processes, such as nanoimprint lithography, photolithography, and atomic layer deposition on CMOS-compatible substrates, must be developed. Integrating topological materials with standard silicon-based platforms is particularly critical to enable hybrid architectures that combine topological protection with conventional logic and memory units. On the application side, topological quantum materials show significant promise in a range of practical device concepts. For instance, in quantum computing, they could be used to construct topologically protected qubits that are inherently robust against local decoherence, reducing the need for complex error correction. In classical electronics, non-reciprocal charge or spin transport enabled by topological edge states could lead to energy-efficient interconnects and signal processing components, such as diode-like conductors or low-power logic gates. In sensing technologies, the extreme sensitivity of topological states to boundary conditions can be exploited for the high-precision detection of magnetic fields, electric fields, or mechanical strain. Furthermore, topological lasers based on non-Hermitian physics could achieve thresholdless lasing and unidirectional light propagation, with applications in integrated photonics. By combining these novel functionalities with advances in scalable fabrication and integration, topological devices could achieve performance improvements in speed, energy efficiency, and fault tolerance, enabling transformative progress in next-generation semiconductor technologies. Ultimately, interdisciplinary collaboration, spanning condensed matter physics, materials science, electrical engineering, and nanofabrication, will be essential to translate topological quantum phenomena from experimental prototypes to reliable, manufacturable technologies with widespread industrial impact.
